# Systemic Resistance to Powdery Mildew in *Brassica napus* (AACC) and *Raphanus alboglabra* (RRCC) by *Trichoderma harzianum* TH12

**DOI:** 10.1371/journal.pone.0142177

**Published:** 2015-11-05

**Authors:** Jawadayn Talib Alkooranee, Yongtai Yin, Tamarah Raad Aledan, Yingfen Jiang, Guangyuan Lu, Jiangsheng Wu, Maoteng Li

**Affiliations:** 1 Department of Biotechnology, College of Life Science and Technology, Huazhong University of Science and Technology, Wuhan, China; 2 Department of Plant Protection, College of Agriculture, University of Basrah, Basrah, Iraq; 3 Hubei Collaborative Innovation Center for the Characteristic Resources Exploitation of Dabie Mountains, Huanggang Normal University, Huanggang, China; 4 Crops Institute, Anhui Academy of Agricultural Sciences, Hefei, Anhui, China; 5 Oil Crops Research Institute, Chinese Academy of Agricultural Sciences, Wuhan, Hubei, China; 6 National Key Laboratory of Crop Genetic Improvement, Huazhong Agricultural University, Wuhan, China; National Key Laboratory of Crop Genetic Improvement, CHINA

## Abstract

*Trichoderma harzianum* TH12 is a microbial pesticide for certain rapeseed diseases. The mechanism of systemic resistance induced by TH12 or its cell-free culture filtrate (CF) in *Brassica napus* (AACC) and *Raphanus alboglabra* (RRCC) to powdery mildew disease caused by ascomycete *Erysiphe cruciferarum* was investigated. In this study, we conducted the first large-scale global study on the cellular and molecular aspects of *B*. *napus* and *R*. *alboglabra* infected with *E*. *cruciferarum*. The histological study showed the resistance of *R*. *alboglabra* to powdery mildew disease. The growth of fungal colonies was not observed on *R*. *alboglabra* leaves at 1, 2, 4, 6, 8, and 10 days post-inoculation (dpi), whereas this was clearly observed on *B*. *napus* leaves after 6 dpi. In addition, the gene expression of six plant defense-related genes, namely, *PR-1*, *PR-2* (a marker for SA signaling), *PR-3*, *PDF 1*.*2* (a marker for JA/ET signaling), *CHI620*, and *CHI570*, for both genotypes were analyzed in the leaves of *B*. *napus* and *R*. *alboglabra* after treatment with TH12 or CF and compared with the non-treated ones. The qRT-PCR results showed that the *PR-1* and *PR-2* expression levels increased in *E*. *cruciferarum*-infected leaves, but decreased in the TH12-treated leaves compared with leaves treated with CF. The expression levels of *PR-3* and *PDF1*.*2* decreased in plants infected by *E*. *cruciferarum*. However, expression levels increased when the leaves were treated with TH12. For the first time, we disclosed the nature of gene expression in *B*. *napus* and *R*. *alboglabra* to explore the resistance pathways in the leaves of both genotypes infected and non-infected by powdery mildew and inoculated or non-inoculated with elicitor factors. Results suggested that *R*. *alboglabra* exhibited resistance to powdery mildew disease, and the application of *T*. *harzianum* and its CF are a useful tool to facilitate new protection methods for resist or susceptible plants.

## Introduction

*Erysiphe cruciferarum* is the ascomycete causative agent of crucifer powdery mildew [1–3], infects a wide range of crucifers, including Chinese cabbage (*Brassica rapa* ssp. *pekinensis*; syn. *B*. *pekinensis*), garden cress (*Lepidium sativum* L.), garlic mustard (*Alliaria petiolata*), African mustard (*Malcolmia africana*), and *Arabidopsis thaliana*, it can infect any above ground part of crucifers and lead to important fluctuations in crop yields by reducing plant growth and the quality and quantity of the seeds [2,4]. *E*. *cruciferarum* has been identified for the first time on *B*. *napus* (AACC) in September 2014 in China [1].

*B*. *napus* (AACC, 2n = 38), an allopolyploid resulting from the natural hybridization between *B*. *rapa* (AA, 2n = 20) and *B*. *oleracea* (CC, 2n = 18) [5], is the main oil seed crop in China. However, the occurrence of powdery mildew disease in *Brassica* crops can lead to heavy yield losses in terms if quantity and quality of seeds by reducing plant growth [6–8]. Thus, exploring new materials that are resistant to powdery mildew disease is important for breeders. The most effective method to control powdery mildew disease is to use resistance genes that are either particular for some fungal pathogens or confer resistance to a wide range of pathogens. These genes cause natural recessive mutations in the Mildew Locus O (*MLO*) gene and provide race non-specific resistance. These genes are effective against nearly all pathogens of powdery mildew [9]. *Raphanus sativus* has the ability to resist different diseases [10, 11]. To transfer the resistance genes of *R*. *sativus* to *B*. *napus*, *R*. *alboglabra* (RRCC, 2n = 34) was constructed through artificial synthesis. Hybridization between *R*. *sativus* (RR, 2n = 16) and *B*. *alboglabra* (CC, 2n = 18) [12, 13], may be a bridge for disease resistance breeding if *R*. *alboglabra* exhibits resistance to powdery mildew, as *R*. *alboglabra* characterization of cross ability and fertility with *R*. *sativus* and five *Brassica* species were investigated by Chen and Wu [14]. In earlier studies, resistance genes have been shown to be involved the defense of various plants against powdery mildew infection. These genes include pathogen-related protein 1 (*PR1*), Beta 1,3 glucanase (*PR2*), Plant defensin 1.2 (*PDF1*.*2*), basic chitinase (*PR3*), pathogenesis-related 4 (*PR4*), and pathogenesis-related 5 (*PR5*) in *Arabidopsis* [15, 16]; one recessive gene *er1* (JI2302), second recessive gene *er2* (JI2480), and *Er3* (IFP13260) in pea [17–19], **P**owdery **M**ildew resistance gene *Pm6* in wheat [20], **P**owdery **M**ildew resistance gene *Pm51* in a Putative Wheat-*Thinopyrum ponticum* Introgression Line [21], or against other disease infection. Moreover, other genes, such as *PR-1*, *PR-2*, *PDF1*.*2*, *PR-3*, Chitinase 620 (*CHI620*), and Chitinase 570 (*CHI570*) in *A*. *thaliana* have exhibited resistance against the bacterial *Pseudomonas syringae* pv. *maculicola* and the fungal pathogen *Colletotrichum higginsianum* [22].

The complex interaction between fungal pathogen and its hosts is a result of the expression of plant defense genes after pathogen infection. Such a relationship either results in development of disease or plant resistance. The success of plant defense against pathogens depends on multiple events involved in the resistance. Moreover, these mechanisms of plant defense are governed by a range of genes singly or synergistically [23]. Some plants express resistance proteins that reveal the presence of specific elicitors, thereby leading to a strong defensive response, which is referred to as elicitor-triggered resistance [24].

Induced resistance (IR) varies according to different signals. Systemic acquired resistance (SAR) and induced systemic resistance (ISR) are two forms of systemic resistance. In both SAR and ISR, plant defenses are released by a previous infection; thus, biotic and abiotic factors play a role in resistance; most agents reduce disease in the infected plants by 20%–85%[25]. ISR is mediated by the *NPR1* gene, which is a key gene involved in disease resistance and phenotypically similar to SAR.

ISR can be defined by induction of defenses in plants against many pathogens via application of plant growth-promoting microorganisms in the soil, as well as direct spreading on plants [26, 27]; whereas SAR is usually caused by a pathogen attack locally. However, the molecular pathways of each systemic resistance are different; ISR depends on two pathways that respond to ethylene and jasmonic acid [28], whereas SAR depends on the salicylic acid (SA) responsiveness [29]. The SA pathway controls the expression of pathogenesis-related (PR) proteins, such as *PR1*, *PR2*, and *PR5*, whereas the JA–ET pathway regulates the expression of a different group of defense genes, i.e., *PR3* and *PDF1*.*2* in *Arabidopsis*; these PR proteins have been defined as *PR2*, *PR5*, *PDF1*.*2*, and basic chitinase (*PR3*) [30].

*Trichoderma* spp. is soil-borne, produces green spores, and is among the ascomycetes that are widespread throughout the world [31]. The fungi of the genus *Trichoderma*, which comprises a group of plant growth-promoting fungi, can colonize the intercellular parts of plant roots and stimulate systemic resistance in all parts of the plant; the actions of these fungi suppress some plant diseases by direct mycoparasitism or antibiosis, as well as indirect induced resistance (IR) [32, 33]. The interaction between *Trichoderma* spp. and plant is correlated with transcriptome and systemic modulations of the plant proteome [33, 34]. *Trichoderma* spp. stimulates ISR through hormonal and molecular pathways in a JA/ET-dependent manner [35, 36]. A recent study has demonstrated that fungi mainly affect the pathogenesis-related genes by increasing their expression levels, thereby resulting in resistance of the treated plants to the leaf pathogens [37].

*T*. *harzianum* is the most effective biocontrol agent against a wide range of plant pathogens [38, 39]. *T*. *harzianum* TH12 isolates are used to produce a cell-free culture filtrate (CF), which has been initially characterized as an elicitor agent of induced systemic resistance to *Sclerotinia* stem rot (SSR) on *B*. *napus* AACC; CF is proven to effectively control pathogen when sprayed onto rapeseed crops [40].

The effects of *E*. *cruciferarum* in *R*. *alboglabra* and *B*. *napus* treated or non-treated with *T*. *harzianum* and its CF have not been well examined. In the present study, we investigated the gene expression of common genes involved in plant disease resistance. The effects of powdery mildew on *B*. *napus* and *R*. *alboglabra* were also investigated in terms of three aspects: first, the effects of *E*. *cruciferarum* on *B*. *napus* and *R*. *alboglabra* in comparison with the non-infected samples at six different time points; second, the effects of *T*. *harzianum* and its CF on *B*. *napus* and *R*. *alboglabra* in comparison with non-treated samples at six different time points; and third, the effects of *E*. *cruciferarum* on *B*. *napus* and *R*. *alboglabra* treated with *T*. *harzianum* and its CF at six different time points of infection, namely, 1, 2, 4, 6, 8, and 10 days post-inoculation (dpi).

## Materials and Methods

### Plant and fungal material

The *R*. *alboglabra* (RRCC), a new material obtained by hybridization of *R*. *sativus* (RR, 2n = 16) and *B*. *alboglabra* (CC, 2n = 18) [12, 13], was provided by Professor Jiangsheng Wu of Huazhong Agricultural University, Wuhan, China. The *B*. *napus* specimen used in this study was stored in our research group laboratory. The *E*. *cruciferarum* isolate HUST-WUH1 (GenBank No. KP730001) used in the inoculations was obtained from a population collected from infected rapeseed plants by our research group in 2015 and was maintained on susceptible rapeseed, Where in October 2014, *B*. *napus* (AACC, n = 19) seedlings in a greenhouse at Huazhong University of Science and Technology, Wuhan, Hubei province of China showed the symptoms typical of powdery mildew in leaves [1]. *T*. *harzianum* TH12 was isolated from a field at College of Life Science and Technology, Huazhong University of Science and Technology [40], and was grown on potato dextrose agar (PDA) for 14 days. In this study, we used *R*. *alboglabra* (RRCC), *B*. *napus* (AACC) seedlings, *E*. *cruciferarum* HUST-WUH1 pathogen and *T*. *harzianum* TH12 as a biotic elicitor, these materials are not endangered or protected species.

### Production of cell-free CF of *T*. *harzianum* TH12

Five mycelial 1 cm-square disks of actively growing TH12 were inoculated with 300 ml of PDB and incubated with a rotary shaker (85 rpm) at 25 ± 2°C. After 20 days, the cell concentration was adjusted to 1.5 × 10^7^ CFU/ml. Pure culture of TH12 was maintained on respective agar slants and stored at 4°C for further use. Cell-free CF from 20-day-old TH12 grown on PDB were prepared by centrifugation (12,000 × g for 15 min), followed by filter sterilization with a 0.4 μm filter unit, and the supernatants were then collected.

### Treatment with inducers and *E*. *cruciferarum* infection

Both genotypes *B*. *napus* and *R*. *alboglabra* were planted in 1000 cm^3^ of 1:1 sand-peat mixture that had been autoclaved twice for 30 min within a 24-h interval. Three seeds of each genotype were sown into each pot, respectively. Both genotypes were grown under greenhouse conditions at 18/14 (± 1)°C (day/night) temperature and a light intensity of 150 μE/m^2^/s^1^ for 12-h light/dark cycles. Irrigation was applied by drenching twice a week. The trifoliate rapeseed seedlings were inoculated with 10 ml suspension of TH12 and its cell-free CF, separately. The plants were treated by soil drenching for one day before inoculation with the pathogen. Conidiophores and conidia of *E*. *cruciferarum* were pressed onto the extended leaves with 1 × 10^6^ conidia /ml (control) and allowed to settle for nearly 20 min. The inoculated rapeseed seedlings were then returned to the greenhouse, whereas the control plants that were un-inoculated were transferred to an independent “clean” greenhouse. Samples were collected at 1, 2, 4, 6, 8, and 10 days post-inoculation (dpi). Each treatment consisted of three replicates, which were used for RNA extraction in liquid nitrogen and stored at –80°C until use.

### Histological assessment of *E*. *cruciferarum* growth

The severity (intensity) of the disease on hosts at 1, 2, 4, 6, 8, and 10 dpi after infected were assessed as follows:

Scale (0–5 scale), given by Khan [41] as: (−) = No infection; (+) = mild infection (25% infection);

(++) = moderate infection, (25%–60% infection) and (+++) = heavy infection, (60%–100% infection).

The seedlings were harvested at indicated time points and prepared for histological test. The investigation focused on the attack sites on randomly selected leaves using an Olympus microscope. The leaves were stained with Trypan Blue, as described previously [42], to examine the fungal structures by light microscopy. The experiment was repeated twice, and 5–10 images were analyzed per time point.

### Gene Expression Assay

The total RNA from the leaves of *B*. *napus* and *R*. *alboglabra* treated or non-treated with inducers at different incubation times (1, 2, 4, 6, 8, and 10 days) was isolated using the RNeasy Plant Mini Kit (Qiagen 74904). After treatment with DNase I (New England M0303S) at 37°C for 30 min, 5 μl of RNA was used for first-strand cDNA synthesis using SuperScript II reverse transcriptase, following the instructions of the manufacturer (Invitrogen 18064–014). All cDNA samples were stored at −20°C until for quantitative real-time polymerase chain reaction (qRT-PCR).

The (qRT-PCR) was performed using Applied Biosystems machines\Step One Software v2.3 (USA). The specific primers used for qRT-PCR are listed in Table 1. PCR reaction was performed using 1 μl of cDNA as templates to a total reaction volume of 20 μl in 0.5 ml PCR tube using iTaq^TM^ SYBR^®^ Green Super-mix with ROX (California, USA). Then, 0.3 μl of each primer and double-distilled H_2_O were added up to a total volume of 8.4 μl and mixed in a 96-cell plate. The *GAPDH* primer was used as control for the experiment. The PCR procedure included pre-denaturing at 95°C for 1 min, followed by 25 and 30 cycles of denaturation at 95°C for 15 s, annealing at 60°C for 15 s, and extension at 72°C for 45 s.

**Table 1:** PCR primers used in the present study.

	Gene description	Primer sequence (5ʹ-3ʹ)
*PR-1*	Pathogenesis-related	Forward: AAAGCTACGCCGACCGACTACGAG
	protein 1	Reverse: CCAGAAAAGTCGGCGCTACTCCA
*PR-2*	Beta 1,3 glucanase	Forward: GTACGCTCTGTTCAAACCGACCC
		Reverse: TTTCCAACGATCCTCCGCCTGA
*PR-3*	basic chitinase	Forward:TCTTTGGTCAGACTTCCCACGAG
		Reverse: GATGGCTCTTCCACACTGTCCGTA
*PDF1*.*2*	Plant defensin 1.2	Forward: TCCATCATCACCCTTCTCTTCGC
		Reverse: CCATGTTGTGCTCCTTCAAGTCG
*CHI620*	Chitinase 620	Forward: AACCAAAATGTTCGTCCCGTCT
		Reverse: GACGCCTACCGTTGCACTCG
*CHI570*	Chitinase 570	Forward: GCTGCCTTCTTCGCTCACGTC
		Reverse: TAGTAGCCCTTTCCTGGTACACA
*GAPDH*	Glyceraldehyde	Forward: CGCTTCCTTCAACATCATTCCCA
	3-phosphate dehydrogenase	Reverse: TCAGATTCCTCCTTGATAGCCTT

### Statistical analysis

The greenhouse experiments were analyzed as completely randomized designs with three replications using the GenStat software, and the means were compared using least-significant difference tests [43].

## Results

### Effects of TH12 and CF drench treatments on systemic resistance to powdery mildew

**Fig 1:**
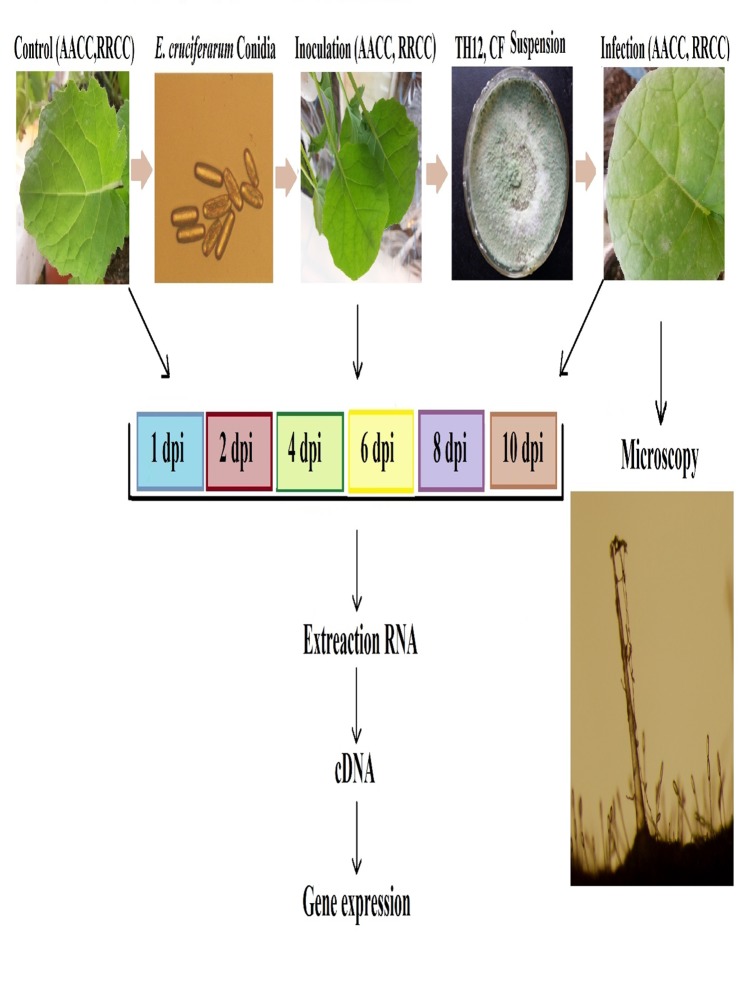
Experimental design and sample collection. 10 ml of TH12 and its cell-free culture filtrate (CF) separately treated by soil drenching in each pot 1 day before treated with 1× 106 conidia / l solution of *E*. *cruciferarum* was used to inoculate the leaf of *B*. *napus* AACC and *R*. *alboglabra* RRCC. Samples were collected to minimize uninfected and infected tissue at 1, 2, 4, 6, 8, and 10 days post-inoculation (dpi). Leafs were used for microscopic analysis of infection stages or pooled for RNA extraction.

*E*. *cruciferarum* is the main pathogen of rapeseed and other crucifers. However, studies describing the process of infection determinants of this obligate ascomycete are limited. In this experiment, we studied the development of powdery mildew disease symptoms and correlated such symptoms with gene expression from a time course of infected *B*. *napus* and *R*. *alboglabra* tissues, representing infection stages from 1 dpi to 10 dpi. In total, our study aims to gain a wide perspective on gene expression in infected and non-infected leaves of susceptible *B*. *napus* and *R*. *alboglabra* (Fig 1).

**Fig 2:**
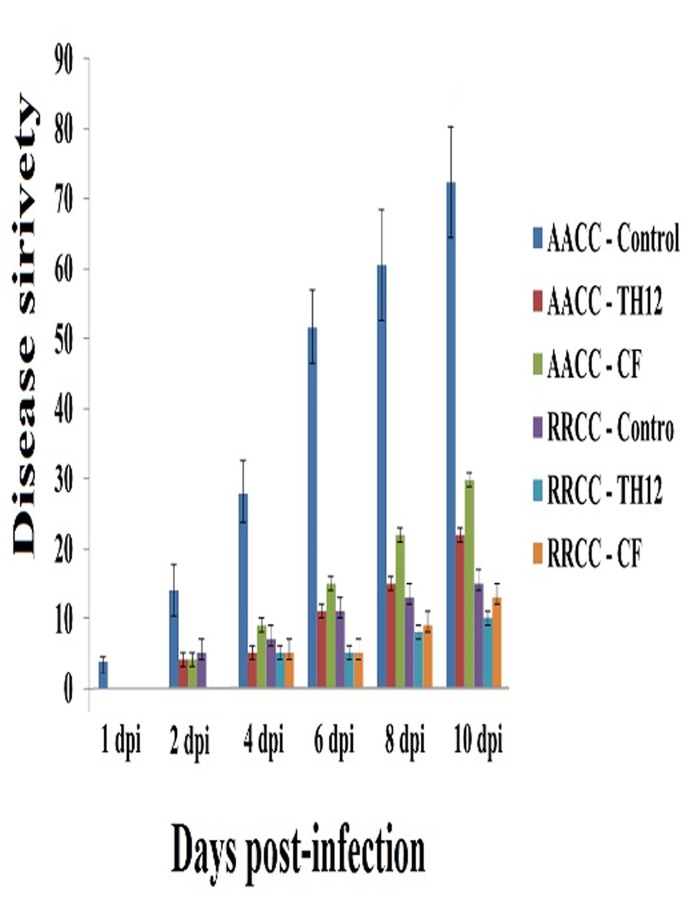
Effects of *T*. *harzianum* TH12 and its cell-free culture filtrate (CF) soil drenches on the development of powdery mildew (*Erysiphe cruciferarum*) on leaves of *B*. *napus* and *R*. *alboglabra* detached from treated rapeseed plants. Disease severity is expressed as white colony at 1, 2, 4, 6, 8 and 10 dpi.

TH12 and CF suppressed the disease severity on rapeseed leaves when applied 1 day before *E*. *cruciferarum* inoculation on *B*. *napus* and *R*. *alboglabra*. The TH12 suspension was the most effective treatment, which suppressed disease severity in *B*. *napus* at 1, 2, 4, 6, 8, and 10 dpi by 0, 4, 5, 11, 15, and 22% respectively. CF suppressed disease severity by 0, 4, 9, 15, 23, and 30% respectively, whereas disease severity in the control reached 4, 14, 28, 52, 61, and 72%, respectively. Both TH12 and CF exhibited significant difference with the control treatment (Fig 2). The TH12 suspension was the most effective treatment, which suppressed disease severity in *R*. *alboglabra* at 1, 2, 4, 6, 8, and 10 dpi by 0, 0, 5, 5, 8, and 10% respectively. CF suppressed disease severity by 0, 0, 5, 5, 9, and 13%, respectively, whereas disease severity in the control reached 0, 5, 7, 11, 15, and 17% at 1, 2, 4, 6, 8, and 10 dpi. These results revealed that both TH12 and CF were significantly different with the control treatment (Fig 2).

### Histological assessment of *E*. *cruciferarum* growth

Similar to other pathogenic powdery mildew and biotrophic fungi, *E*. *cruciferarum*, is non-cultural on standard laboratory media, and reproduces and proliferates only on susceptible crucifer hosts. After inoculating the upper leaf surface with conidia of *E*. *cruciferarum*, samples were collected at different time periods for histological examination. Approximately four leaves from all infected plants of each genotype were examined for each time point.

**Fig 3:**
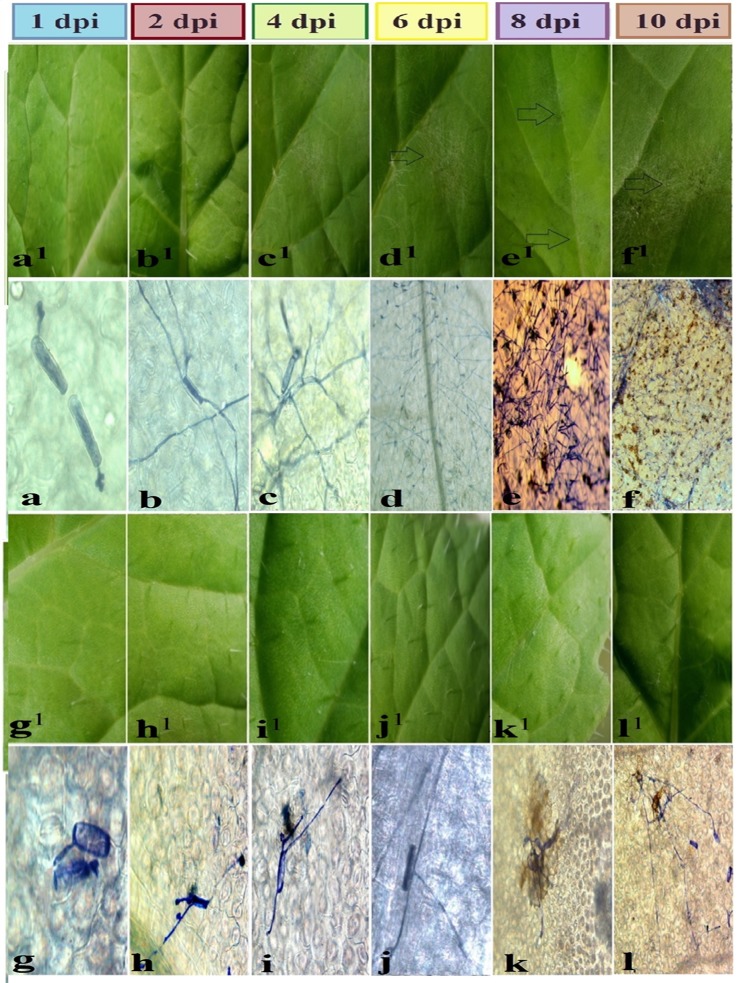
Symptoms of *Erysiphe cruciferarum* on leaves infected of *B*. *napus* of 6 time points. Symptom images were (a^1^, b^1^, c^1^, d^1^, e^1^, f^1^) and light micrograph were (a, b, c, d, e, f) for leaves of *R*. *alboglabra* infected by pathogen were (g^1^, h^1^, i^1^, j^1^, k^1^, l^1^) and light micrograph were (g, h, i, j, k, l) for leaves of RRCC infected by pathogen at 1, 2, 4, 6, 8 and 10 days post-inoculation (dpi) respectively. Stocks indicate to colonies and the growth of pathogenic fungus. Scale bars for light micrograph at 8 and 10 dpi are 25 μm.

At 6 dpi, symptoms of infection were observed in white powdery mildew on the upper surface of *B*. *napus* leaves. Symptoms can be observed with the naked eye and increased with the progress of time (Fig 3D^1^, 3E^1^, and 3F^1^). The observable signs of the pathogen included hyphae, conidia, conidiophores, and dead cells on the infected leaf surface, whereas visual symptoms were not observed in the *R*. *alboglabra* genotype (Fig 3G^1^–3I^1^). At 1 dpi, the structures of pathogenic fungi appeared on the securities under microscopic examination, but lower compared with *B*. *napus* (Fig 3G). Microscopic examination showed the conidia of the pathogenic fungus produced germination tubes on the leaves of both genotypes, where conidia produced primary germ tube, aspersorium, and primary hyphae at 2 dpi, followed by hyphae in *B*. *napus* and to lesser extent in *R*. *alboglabra* (Fig 3B). The rapid growth and wide spread of the pathogen and hyphae were also observed. Several conidia were observed on the upper surface of the infected leaves of *B*. *napus*. The fungus growth increased with time (Fig 3C–3F). On the contrary, the microscopic examination on *R*. *alboglabra* showed that most of the conidia produced germination tubes, but failed to format the hyphae. These conidia did not develop intensively and rapidly compared with *B*. *napus* during the same time period (Fig 3I–3l). The results also showed the presence of dead cells in *B*. *napus*, and *R*. *alboglabra* increased. However, fungus growth was densely spread and increased with time. The microscopic examination clearly emerged after 6, 8, and 10 dpi (Fig 3F and 3I). The percentage of spores that successfully germinated on the leaf surface did not differ between the genotypes and time points. However, the further development of the fungus differed during cell penetration; development was lower for *R*. *alboglabra* (5%) compared with *B*. *napus* (75%) at 2 dpi. This continued penetration resistance reached 14% successful penetration at 4 dpi compared with 68% of *B*. *napus* (Fig 3G and 3H). Thus, no or very little colonies were formed on *R*. *alboglabra* at 4, 6, 8, and 10 dpi. Finally, the histological study confirmed that the *R*. *alboglabra* genotype was excluded from infection because it was able to prevent the pathogenic fungus *E*. *cruciferarum* from penetrating the cell wall of host successfully and inhibited the growth of the pathogen at different stages. This penetration resistance was activated at 1 dpi and allowed complete resistance under certain conditions.

### Expression levels of gene-related pathogen of healthy and infected *B*. *napus* and *R*. *alboglabra* leaves by powdery mildew

The disease is associated with the expression of disease resistance genes in plant. To determine the ability of *B*. *napus* and *R*. *alboglabra* to resist *E*. *cruciferarum* infection during different time periods (1, 2, 4, 6, 8, and 10 dpi), we investigated the expression profile of defense-related genes in plants by qRT-PCR. Six primers for each genotype were used (Table 1), including *PR-1*, *PDF1*.*2*, *PR-2*, *PR-3*, *CHI620*, and *CHI570* genes. The housekeeping gene, *GAPDH*, was used as reference gene. For both genotypes, the *PR-1* and *PR-2* genes were used as markers for the SA signal pathway, whereas the *PDF1*.*2* gene was used as a marker for the JA/ET signal pathway, as well as the putative marker genes *PR-3*, *CHI620* and *CHI570*.

**Fig 4:**
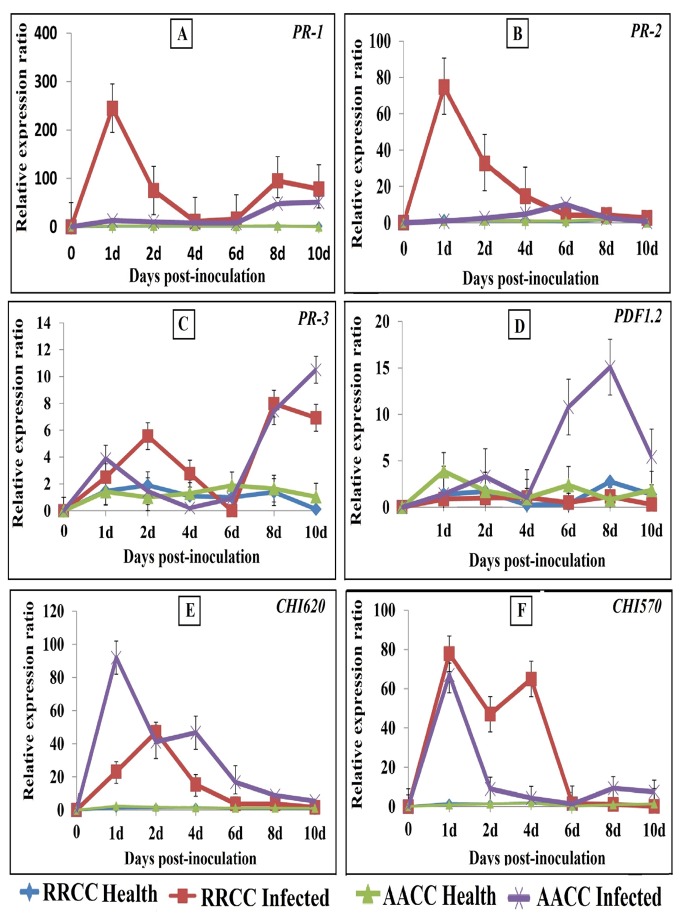
Expression of defense-related genes in three potted for each time of *B*. *napus* and *R*. *alboglabra* genotypes of 6-weeks-old inoculated by pressing diseased leaves by powdery mildew onto leaves. Leaves were collected 1, 2, 4, 6, 8 and 10 days post infection. Total RNA was extracted, and cDNA was synthesized. Expression levels of the *PR-1*, *PR-2*, *PDF1*.*2* (glucanase; BGL2), *PR-3* (basic chitinase), *CHI620* and *CHI570* (chitinase) genes were monitored by RT q- PCR. The expression levels of genes were compared with the expression level of *GAPDH*.

The results indicated that most of the genes were amplified (90.0%) after *E*. *cruciferarum* infection. The gene expression increased over time in both genotypes, which was higher in plants inoculated with the fungus pathogen compared with the healthy plants.The gene expression levels increased in *R*. *alboglabra* infected compared with non-infected and with *B*. *napus*, its showed increased gene expression levels of *PR1* and *PR2* were up-regulated by 218.86-fold and 43.08-fold and at 1 dpi, respectively, compared with an average of 2-fold increased expression in non-infected (healthy) plants (Fig 4A). The expression levels of the *PR-1* and *PR-2* genes increased in *B*. *napus* infected were up-regulated by 69.05-fold at 8 dpi and 6.14-fold at 6 dpi, respectively, compared with an average of 1.2-fold increased expression in non-infected plants (Fig 4B). The *PR-3* and *PDF1*.*2* genes were used as markers for the JA/ET signal pathway. The expression of the *PR-3* gene increased and peaked 10.10-fold (1.04 –to 10.50-flod) at 10 dpi in infected *B*. *napus* were up-regulated by 67.77-fold (from 0.117- to 7.93-fold) at 8 dpi in infected *R*. *alboglabra*, respectively, compared with then non-infected ones (Fig 4C). The expression levels of the *PDF*.*1*.*2* gene showed significant differences between infected and non-infected plants were up-regulated by 8.151-fold (from 1.85- to 15.07-fold) at 8 dpi in *B*. *napus* infected (Fig 4D). We also studied other defense-related genes to investigate the comparison between the signal transduction pathways in both genotypes. Thus, we used qRT-PCR to analyze the effects of different times and infections on the expression of the following putative marker genes: *CHI620* and *CHI570* (chitinase), which are anti-pathogenic enzymes. The expression levels of *CHI620* gene increased clearly with time and reached its peak at 1 dpi were up-regulated by 58.62 -fold (from 1.64- to 96.15-fold) in *B*. *napus* and reached 37.29-fold (from 1.21- to 45.13-fold) at 2 dpi in *R*. *alboglabra* infected compared with the non-infected (healthy) ones (Fig 4E). The expression levels of the *CHI570* gene increased clearly with time and reached its peak at 1 dpi in *B*. *napus* and in *R*. *alboglabra* than in non-infected (healthy), which were up-regulated by 40.86-fold (from 1.64- to 66.73-fold) and 59.87-fold (from 1.34- to 80.23-fold), respectively (Fig 4E).

### Effects of TH12 and CF on the gene expression levels of non-infected *B*. *napus* and *R*. *alboglabra* leaves

We determined the expression profile of defense-related genes in *B*. *napus* and *R*. *alboglabra* treated or un-treated with TH12 or CF without infection by powdery mildew during different time periods (1, 2, 4, 6, 8, and 10 dpi) by quantitative real time-polymerase chain reaction (qRT-PCR). Six primers were used (Table 1) to find out the pathways of resistance of *R*. *alboglabra*.

**Fig 5:**
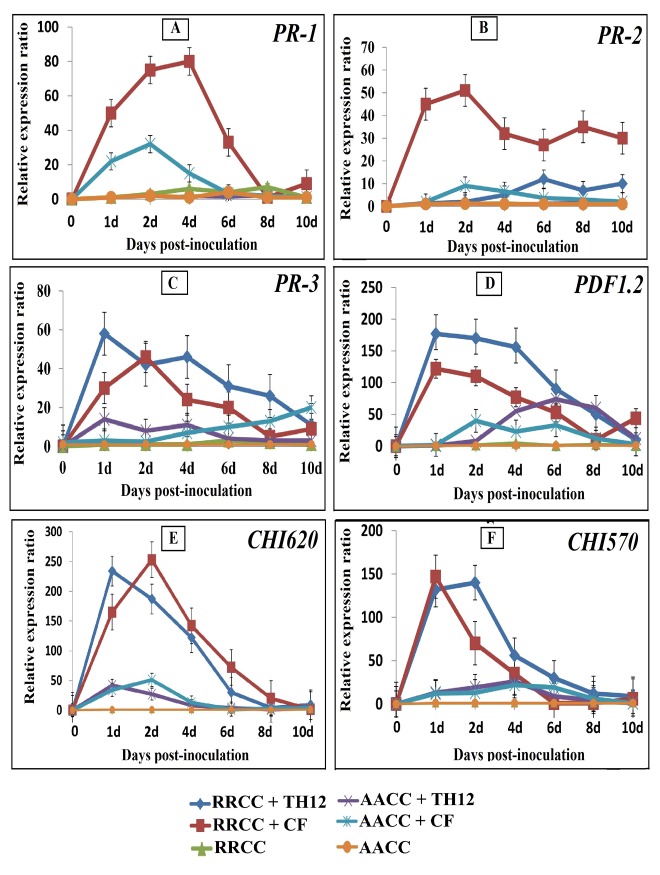
Expression of defense-related genes of *B*. *napus* and *R*. *alboglabra* genotypes of 6-weeks-old inoculated with suspensions 10 ml of TH12 and its cell-free culture filtrate (CF) separately treatedby soil drenching. Three potted for each time non-inoculated served as control plants. Leaves were collected 1, 2, 4, 6, 8 and 10 days post inoculation. Total RNA was extracted, and cDNA was synthesized. Expression levels of the *PR-1*, *PR-2*, *PDF1*.*2* (glucanase; BGL2), *PR-3* (basic chitinase), *CHI620* and *CHI570* (chitinase) genes were monitored by RT q- PCR. The expression levels of genes were compared with the expression level of *GAPDH*.

The expression levels of *PR-1* peaked at 2 dpi in *B*. *napus* treated with TH12 and up-regulated by 1.34-fold (from 1.60- to 2.15-fold) and up-regulated by 17.91-fold (from 1.60- to 28.67-fold) at 2 dpi when treated with CF *T*. *harzianum* TH12 compared with non-treated ones (Fig 5A). Moreover, the expression levels also increased in *R*. *alboglabra* treated with CF, where the *PR-1* gene was up-regulated by 21.92 -fold (from 3.7- to 81.12-fold) at 4 dpi than with the non-treated ones (Fig 5A).

The expression levels of the PR-2 gene were increased when treated with CF in both genotypes, followed by treatment with TH12, compared with the non-treated ones. The expression levels of *PR-2* peaked at 2 dpi in *B*. *napus* treated with CF and were up-regulated by 7.47-fold (from 0.85- to 9.35-fold), whereas these were up-regulated by 31.17-fold (from 1.73- to 53.94-fold increase) at 2 dpi in *R*. *alboglabra* treated with CF (Fig 5B).

The results indicated that most of the amplified genes were affected by a combined inoculation with TH12 or CF. TH12 and CF influenced the resistance genes and showed strong effect on the *PR-3* and *PDF1*.*2* genes in treated plants than that in non-treated ones. The expression level of the *PR-3* genes were up-regulated by 6.57-fold (from 2.39- to 15.72-fold) peaked at 1 dpi in *B*. *napus* treated with TH12, and were up-regulated by 8.97-fold (from 2.39- to 21.46-fold) at dpi when treated with CF (Fig 5C). The expression level of the *PR-3* genes also increased in *R*. *alboglabra* when treated with TH12, which peaked at 1 dpi and up-regulated by 34.62-fold (from 1.74- to 60.25-fold) and at 2 dpi and up-regulated by 25.93-fold (from 2.11- to 54.72-fold) when treated with CF, compared with the non-treated ones (Fig 5C). The expression of *PDF1*.*2* gene in *B*. *napus* treated with TH12 and CF were up-regulated by 25.03-fold (from 2.92- to 73.10-fold) at 6 dpi and 20.31-fold (from 1.83- to 37.17-fold) at 2 dpi, respectively, compared with the non-treated plants (Fig 5D). The expression of *PDF1*.*2* gene also increased in *R*. *alboglabra* when treated with TH12, which peaked at 1 dpi and up-regulated by 81.69-fold (from 2.13- to 174-fold), whereas at 1 dpi and up-regulated by 57.67-fold (from 2.13- to 122.85-fold) when treated with CF, compared with the non-treated ones (Fig 5D).

The expressions levels of *CHI620* and *CHI570* genes increased in *B*. *napus* treated with TH12 at 1 and 4 dpi and were up-regulated by 20.46-fold (from 1.98-fold to 40.53-fold increase) and 12.87-fold (from 1.82-fold to 23.44-fold increase), respectively (Fig 5E and 5F). These were up-regulated by 40.26-fold (from 1.18-fold to 47.51-fold increase) and 12.22-fold (from 1.82-fold to 22.25-fold increase) at 1 dpi in *B*. *napus* treated with CF, respectively, compared with the non-treated plants (Fig 5E and 5F). The expression levels of *CHI620* and *CHI570* genes increased in *R*. *alboglabra* treated with TH12, reaching 59.62-fold (from 2.24-fold to 133.57-fold) and 142.28-fold (from 1.02-fold to 145.13-fold increase) at 1 dpi, respectively (Fig 5E and 5F). In addition, the expression levels of *CHI620* and *CHI570* genes increased in *R*. *alboglabra* treated with CF, which reached 209.55-fold at 2 dpi and 107.58-fold at 1 dpi, respectively (Fig 5E and 5F). The expression levels of all the genes remained very low in the non-treated genotypes, and expression levels differed among genes because of the effect of the elicitor on genotypes.

### Effect of drenching TH12 and CF treatments on the gene expression levels upon powdery mildew infection

**Fig 6:**
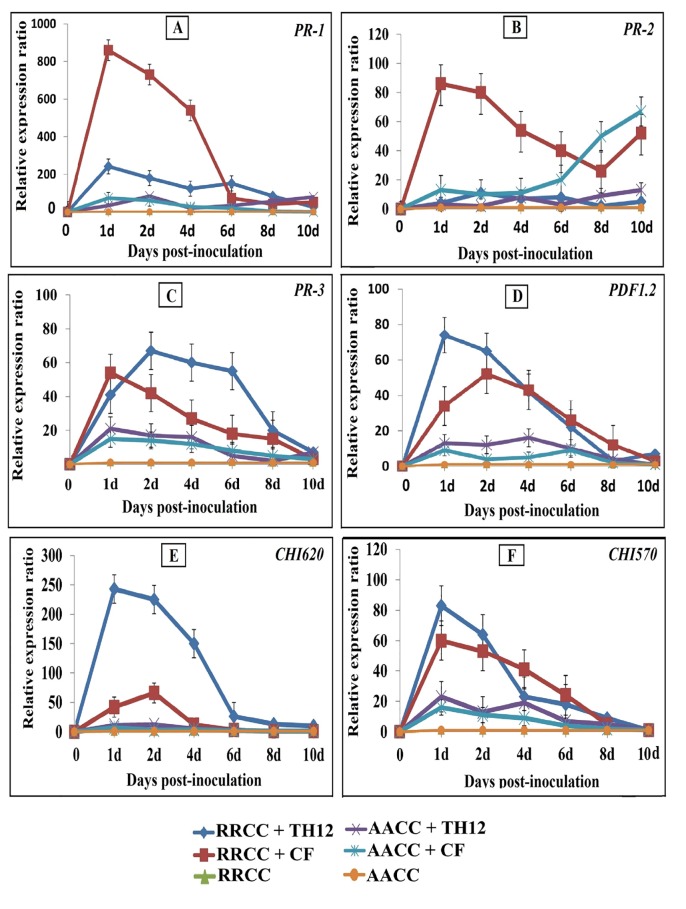
Expression of defense-related genes of *B*. *napus* AACC and *R*. *alboglabra* RRCC genotypes of 6-weeks-old inoculated with suspensions 10 ml of TH12 and its cell-free culture filtrate (CF) separately treatedby soil drenching 1 day before infection by pressing diseased leaves by powdery mildew onto leaves. Three potted for each time non-inoculated served as health plants. Leaves were collected 1, 2, 4, 6, 8 and 10 days post infection. Total RNA was extracted, and cDNA was synthesized. Expression levels of the *PR-1*, *PR-2*, *PDF1*.*2* (glucanase; *BGL2*), *PR-3* (basic chitinase), *CHI620* and *CHI570* (chitinase) genes were monitored by qRT-PCR. The expression levels of genes were compared with the expression level of *GAPDH*.

We determined the expression levels of the gene-related pathogens in *B*. *napus* and *R*. *alboglabra* infected by *E*. *cruciferarum* 1 day after treatment with TH12 and CF. The gene expression levels were higher in the treated plants compared with non-treated plants at 1, 2, 4, 6, 8, and 10 dpi. Plants treated with CF suspension showed greater effect, wherein the *PR-1* and *PR-2* gene expression levels were up-regulated by 419.83-fold (from 1.7-fold to 836.12-fold) and 45.52-fold (from 1.9-fold to 86.5-fold) at 1 dpi, respectively, in *R*. *alboglabra* infected compared with the non-treated plants, The CF treatment had a weaker effect on *B*. *napus* infection (Fig 6A and 6B). The *PR1* gene expression levels in infected *B*. *napus* treated with CF were up-regulated by 14.28-fold (from 1.4-fold to 46.20-fold) at 1 dpi compared with non-treated (Fig 6A). The *PR-2* gene expression levels in infected *B*. *napus* treated with CF were up-regulated by 27.68-fold (from 2.3-fold to 63.67-fold) at 10 dpi compared with non-treated (Fig 6B). The TH12 treatment had the weakest effect, affecting *PR-1* and *PR-2* genes in *R*. *alboglabra* infected were up-regulated by 129.41-fold at 1 dpi and 10.96-fold at 2 dpi, respectively (Fig 6A and 6B). TH12 treatment affected on expression levels *PR-1* and *PR-2* in *B*. *napus* were up-regulated by 20.41-fold and 8.52-fold at 2 dpi, respectively, compared with the non-treated ones (Fig 6A and 6B).

The expression levels of *PR-3* and *PDF1*.*2* genes significantly increased after treatment with TH12 in *R*. *alboglabra* infected leaves were up-regulated by 38.70-fold at 2 dpi and 66.23-fold at 1 dpi compared with the non-infected plants, the expression levels of *PR-3* and *PDF1*.*2* genes in *R*. *alboglabra* infected treated with CF were up-regulated by 49.29-fold at 1 dpi and 38.86-fold at 2 dpi, respectively (Fig 6C and 6D). The expression levels also increased in *B*. *napus* infected leaves treated with TH12 were up-regulated by 15.43-fold at 1 dpi and 8.81-fold at 4 dpi increase, respectively compared with the non-infected leaves (Fig 6C and 6D). The CF treatment had the weakest effect on the expression levels of the *PR-3* and *PDF1*.*2* genes in *B*. *napus* were up-regulated by 11.30-fold and 4.73-fold at 1 dpi, respectively, compared with the non-treated ones (Fig 6C and 6D).

The expression levels of the *CHI620* and *CHI570* genes increased in *R*. *alboglabra* after treatment with TH12 were up-regulated by 33.07-fold and 50.43-fold at 1 dpi, whereas after treatment with CF reached 32.030- and 49.55-fold at 1 dpi, respectively, compared with the healthy plants (Fig 6E and 6F). In *B*. *napus* plants treated with TH12, the expression levels of the *CHI620* and *CHI570* genes reached (6.23-fold and 12.87-fold at 1 dpi), whereas those treated with CF reached 3.79-fold and 8.99-fold at 1 dpi compared with the non-treated ones. SA and the JA/ET signaling pathways combined to signal ISR against powdery mildew after treatment with TH12 and its CF in *B*. *napus* and *R*. *alboglabra* (Fig 6E and 6F).

## Discussion

We have demonstrated that a genotype *Raphanus alboglabra* (RRCC) showed resistance against *Erysiphe curcuferarum* at early time through histological and genetic examinations, and we have demonstrated that localized pre inoculation with a virulent pathogen and application of the biotic elicitor *Trichoderma harzianum* TH12 and its cell-free culture filtrate (CF) effectively enhances resistance of *Brassica napus* (AACC) and *R*. *alboglabra* (RRCC) to the fungal pathogen *E*. *curcuferarum*. Importantly, we show that enhanced resistance to both pathogens displays the hallmarks of typical ISR or SAR responses, including the accumulation of resistance genes transcripts and requirement for SA or JA/ETH pathways.

The *B*. *napus* genotype tested in this study was susceptible to *E*. *cruciferarum*. However, histological measurements showed *E*. *cruciferarum* was received reluctance to penetrate in *R*. *alboglabra*. This resistance was active from penetration until 24 h after infection. *E*. *cruciferarum* was advanced colonization on the leaves of its host. Young hyphae at the inoculation sites can be observed under a microscope at 2 dpi in the AACC genotype. Furthermore, typical and macroscopic hyphae around the inoculation areas could be observed with the naked eyes at about 6 dpi, whereas visual symptoms were not observed in the *R*. *alboglabra* genotype. Thus, we found that colony formation was a rare case at 1–8 dpi, and no visual symptoms were disclosed in *R*. *alboglabra* compared with *B*. *napus*.

Although the cellular mechanisms responsible for colony abortion remain unknown, the presence of different mechanisms, such as callose apposition, hypersensitive response, and protein cross linking, can lead to abortion of colony; results were obtained by using a resistant pea er1 gene compared with a susceptible check [19].

Resistance to *E*. *cruciferarum* has been histologically and genetically characterized previously [44, 45]. Previous studies have shown that *Brassica* spp. genotypes are susceptible to *E*. *cruciferarum*, whereas various resistance traits were was observed in *R*. *sativus*, B. *rapa*, and *B*. *oleracea* [46]. A similar phenomenon was observed in the interactions between powdery mildew fungi and other non-hosts [13, 47].

*R*. *alboglabra* (RRCC) is a hybrid of *R*. *sativus* (RR) and *B*. *alboglabra* (CC), its ability of resistance to the powdery mildew disease may be due to the high response to identify the pathogen during the early stages of infection. The first critical stages in host after germination of tube is the response to defend, that lead to failed growth of hyphae through the inhibition of the formation of appressorium by production of cell wall appositions (papillae), which are thought to represent a chemical anti-microbial blockade and physical deployed to infection and which are composed of an apparently amorphous mixture of pectin, cellulose, callose, lignins, silicon, phenolics, H2O2, fungal enzyme inhibitors and antimicrobial metabolites (phytoalexins) [48, 49]. In addition other responses associated with the infection such as induction of several elicitor-response genes and reactive oxygen intermediates (ROI) production [50], and its resistance to diseases may be attributed to the presence of various plant antifungal proteins that are believed to be involved in plant protection through growth inhibition of pathogens, such as *Rs*-AFP1 and *Rs*-AFP2 in *R*. *sativus* (RR) [51, 52], and through the production of high peroxidases, which have important roles in plant protection because they reduce pathogenic attack [53], other strong defense responses in RRCC mesophyll cells, including reactive oxygen species (ROS) accumulation, callose deposition (1,3-β-glucan cell wall polymer), and post-penetration cell death or post haustorial (HR). The presence of these defensive responses to powdery mildew infection is often observed in other non-host interactions [54–56].

The extracted defense-related protein samples from *R*. *sativus* inoculated with *A*. *alternata* and *Fusarium oxysporum* pathogens showed significant inhibition of fungal pathogen growth compared with the non-inoculated sample; *PR2* (glucanase) and *PR3* (chitinase) proteins are found in infected plants [57]. *B*. *oleracea* juice is effective against certain pathogenic fungi; it reduced the appearance of germ tubes of *Candida albicans* and inhibited the growth of blastoconidia [58].

To determine whether *R*. *alboglabra* and *B*. *napus* resist powdery mildew disease systemically with or without *T*. *harzianum* TH12 or its CF treatment, we studied the expression profile of pathogenesis-related genes in plants by real time quantitative-polymerase chain reaction (qRT–PCR). Gene expression was examined on the selected genotype at 1, 2, 4, 6, 8, and 10 dpi. We used the *PR-1/ PR-2* and *PR-3/ PDF1*.*2* genes as markers of dependent signal transduction pathway for the SA and JA/ET in both genotypes, respectively [59–61]. Six genes have been selected because of their ability to defend plants against powdery mildew after being stimulated by biotic elicitors or a biotic elicitor and attack of different pathogens (17; 22). One housekeeping gene was also included.

The plant hormones SA, JA, and ET have been implicated with defense transduction in separate pathways [30]. Biotrophic and hemi-biotrophic pathogens are more sensitive to pathway SA-dependent responses, whereas herbivorous insects and necrotrophic fungus are commonly deterred by JA/ET-dependent pathway defense [62, 63]. The genes *PR-1*, *PR-2* and *PR-5* required SA signaling for pathogen-induced activation, whereas the genes *PDF1*.*2*, *PR-3* and *PR-4* are induced by pathogens through JA-dependent and ET-independent pathways [22].

We found a significant increase in the gene expressions of *PR-1* and *PR-2* in 1 and 2 dpi in *R*. *alboglabra* compared with the gene expression in *B*. *napus*. Such increases in gene expression occurred to counteract biotrophic pathogen, thereby suggesting that RRCC quickly responds to infection by developing resistance to *E*. *cruciferarum*, and resistance continues to increase at 2–4 dpi. The elicitor is biotrophic for SA pathway, thereby stimulating *PR-1* and *PR-2* genes rapidly. Plant resistance to biotrophic pathogens is often coupled with stimulation of defense responses by the SA pathway [64].

The expressions of *PDF 1*.*2* and *PR-3* increased quickly at 8 dpi because these genes depend on the JA/ETH pathway and the biotrophic elicitor cannot induce the JA/ETH pathway, but increased expression levels of these genes in the treated plants with *T*. *harzianum* TH12 and its CF in both genotypes compared with healthy plants. The JA-dependent pathway contributed to resistance against necrotrophic pathogens that are antagonistic to the SA-dependent pathway [64, 65]. We further confirmed the important contribution of the SA signaling pathway in *R*. *alboglabra* to resistance against *E*. *cruciferarum* at the site of infection (locally). The increase in SA levels leads to systemic and local expressions of a subset of pathogen-related genes (PR-genes), also known as systemic acquired resistance (SAR) genes [66, 67]. In addition, the expressions of marker genes *CHI620* and *CHI750* were significant in both genotypes at 1 and 2 dpi. *B*. *napus* plants exhibited SAR after being infected with foliar pathogens through activation of *PR-1* and *PR-2* genes; high levels of these genes have been detected at 1 dpi [68].

Published reports have analyzed the gene expression in biotrophic pathogens, and have indicated that optimization of the process sampling techniques is the key to maximizing the tissue of the pathogen compared with the host, especially in the early stages of infection [69]. The analysis showed that powdery mildew inoculation, time, and genotype are the key factors controlling gene expression. As expected, given the differences among genotypes and between inoculated and healthy leaves, the transcription profiles for some genes provided different results. Resistance to diseases is associated with the expression of disease-related genes that are markers of resistance. The presence of dead cells is due to hypersensitivity, which in turn results from reducing the infection. These dead cells are clearly present after 4 dpi, but this defense response is not sufficient to prevent the growth of pathogenic fungi.

Numerous studies have shown that *Brassica* sp. can inhibit the growth of some foliar pathogens (fungal and bacterial; Gram-positive or Gram-negative) may return because of the existence of genes controlling the production of enzymes such as *BjCHI1*, which is a plant chitinase gene in *B*. *juncea* [70, 71]. Three chitinase genes, namely ***Br*** *assica* **C**hitinase **l**ike **P**rotein (*BrCLP1*, *BrCLP2*, and *BrCLP3*) may be involved in *Brassica* resistance against biotic stresses in Chinese cabbage against *Pectobacterium carotovorum* subsp. *carotovorum* [72] (Ahmed *et al*., 2012). Biotic elicitors in plant roots play a role in improving plant health. ISR and plant growth promotion are the important mechanisms by which selected mycorrhizal fungi and plant growth-promoting rhizobacteria in the rhizosphere prime the entire plant, thereby promoting defense against a broad range of pathogens [73]. CF of *T*. *asperellum* STK-1 can induce systemic resistance in *A*. *thaliana* and can decrease lesion development and growth of the pathogen *P*. *syringae* pv. *tomato* DC3000 [74].

*T*. *harzianum* T39, when applied 48−72 h before inoculation, reduces downy mildew disease by approximately 63% on susceptible grapevine cultivars; downy mildew was caused by *Plasmopara viticolain* under greenhouse conditions[75].

## Conclusion

Our results showed that infection of *B*. *napus* and *R*. *alboglabra* is activated by the SA signaling pathway at the early stage of infection and by the JA/ET signaling pathway at the later stage of infection. Infection is highly activated by chitinase in genotypes. The timing of activation of these signaling pathways may be essential for the development of plant resistance to pathogenic infections. Meanwhile, CF of TH12 elicited ISR against powdery mildew through multiple pathways in *B*. *napus* and *R*. *alboglabra* plants.
